# Intraoperative contrast-enhanced ultrasound features of progressive multifocal leukoencephalopathy: a case report

**DOI:** 10.3389/fnimg.2026.1842218

**Published:** 2026-05-18

**Authors:** Lapo Bonosi, Giovanni Tringali

**Affiliations:** Department of Neurosurgery, ARNAS Civico Di Cristina Benfratelli Hospital, Palermo, Italy

**Keywords:** CEUS, contrast-enhanced ultrasound, demyelinating disease, diffuse intrinsic brain lesion, IOUS, progressive multifocal leukoencephalopathy

## Abstract

We report the intraoperative ultrasound findings in a male patient with history of stage IVb mantle cell lymphoma who developed new-onset personality changes and gait instability. Brain imaging revealed an extensive right frontal white matter lesion with subtle multifocal spreading. Given the absence of contrast enhancement on MRI and considering the high-dose steroid therapy administered, an open biopsy was performed due to suspicion of cerebral lymphoma. During procedure, intraoperative ultrasound with and without microbubble contrast was carried out, demonstrating distinct echotexture features, determining the site of biopsy. The histological analysis led to a diagnosis of progressive multifocal leukoencephalopathy (PML). To the best of our knowledge, this may represent one of the first descriptions of intraoperative ultrasound characteristics in PML.

## Introduction

Progressive multifocal leukoencephalopathy (PML) is a rare demyelinating disease of the central nervous system (CNS) caused by reactivation of the ubiquitous John Cunningham virus (JCV). It primarily affects immunocompromised individuals, including patients with HIV infection, hematologic or lymphoproliferative malignancies, and those receiving iatrogenic immunosuppression, particularly with newer immunotherapies. Accurate epidemiological data are limited; however, the overall incidence of PML has been estimated at approximately 0.11 per 100,000 person-years. Despite its rarity, PML carries a substantial clinical burden, with high mortality rates (20%−80%) and severe neurological sequelae among survivors (Joly et al., 2023; Adang and Berger, 2015). Standardized diagnostic criteria remain incompletely defined (Cinque et al., 2003), and although detection of JCV DNA in cerebrospinal fluid (CSF) or characteristic histopathological findings in combination with compatible clinical and radiological features is widely considered diagnostic, misdiagnosis has been reported (Mousa et al., 2023).

In this context, we believe it is important to share with the scientific community one of the first ultrasound descriptions of this rare condition.

## Case report

**Figure 1:**
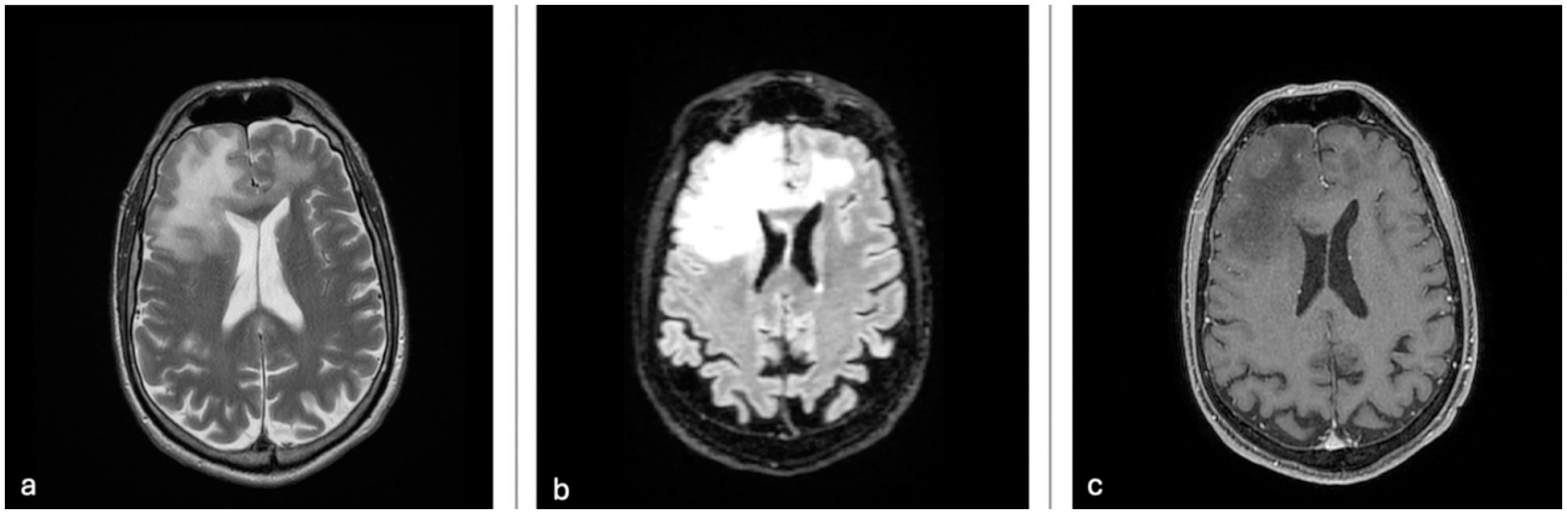
**(a)** Axial T2-weighted MRI; **(b)** Axial CUBE-FLAIR MRI; and **(c)** Axial post-gadolinium 3D-T1 weighted FSPGR MRI. It is possible to appreciate the diffuse pattern of right frontal lobe white matter infiltration with cortical sparing and the complete absence of contrast uptake. The mass effect exerted on the surrounding brain parenchyma is minimal.

We report the case of a 66-year-old Caucasian man with a history of stage IVB mantle cell lymphoma treated with multiagent immunochemotherapy (CHOP-R and R-DHAP, three cycles each), followed 6 months later by autologous stem cell transplantation (ASCT) using mobilized peripheral blood and a FEAM conditioning regimen. The patient achieved complete remission, with no evidence of disease at 2-year follow-up. After more than 2 years of disease-free survival, he was referred to our institution because of the subacute onset of altered mental status and gait disturbance. Upon closer neurological examination, the patient presented with visual disturbances described as flashing lights when moving his eyes upward, as well as behavioral and cognitive changes such as ideomotor apraxia and a mild left lower limb weakness. Contrast-enhanced brain magnetic resonance imaging (MRI) demonstrated extensive multifocal subcortical T2/FLAIR hyperintensities, most prominent in the right frontal lobe, with contralateral extension through the corpus callosum and no gadolinium enhancement (Figure 1).

**Figure 2:**
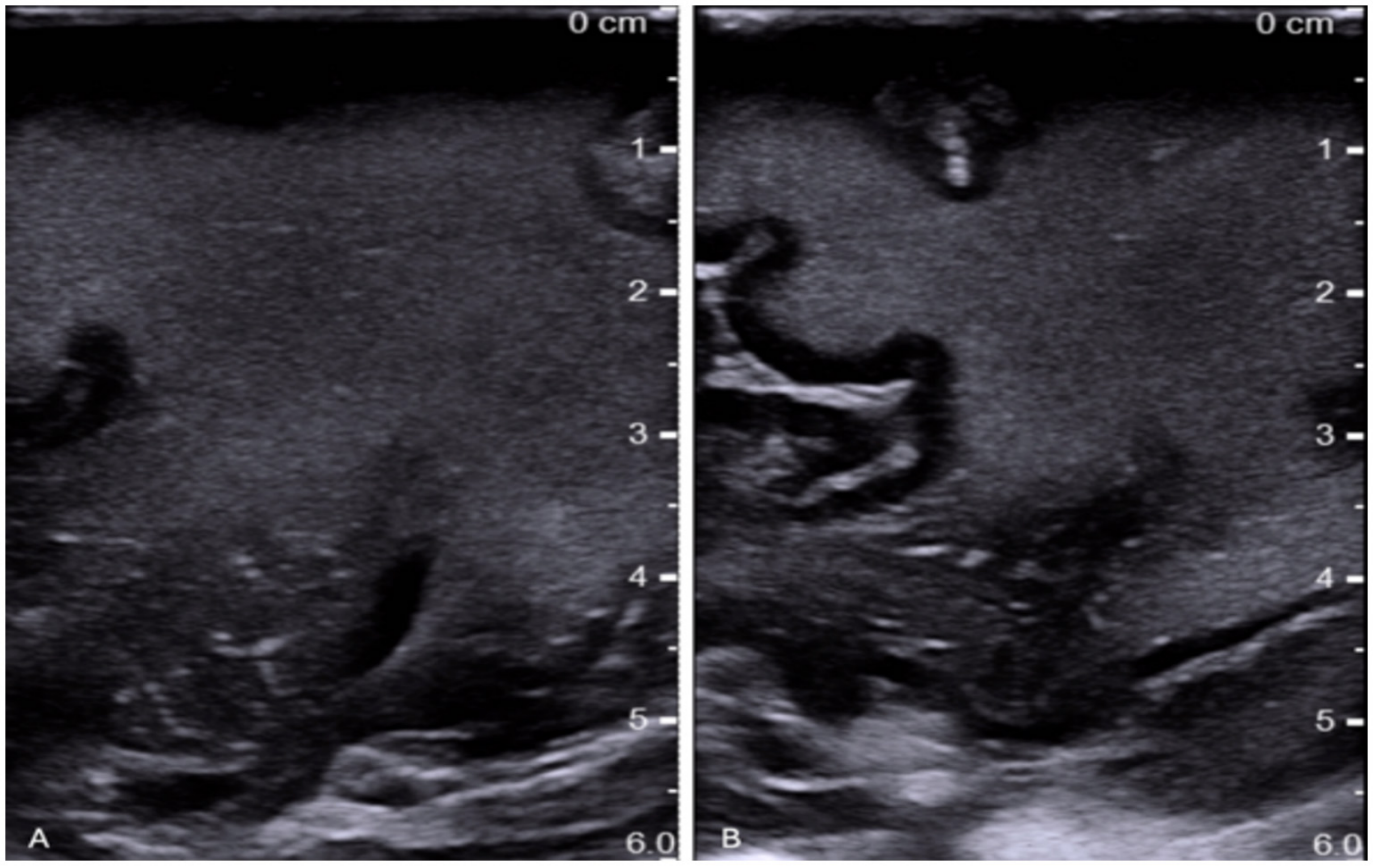
**(A)** we visualized primarily the lesion, characterized by blurred margins and a hyperechoic appearance compared to the normal echogenicity of the cerebral parenchyma. **(B)** we demonstrated the almost exclusive involvement of the subcortical white matter, with preservation of the sulci and cortical gray matter.

**Figure 3:**
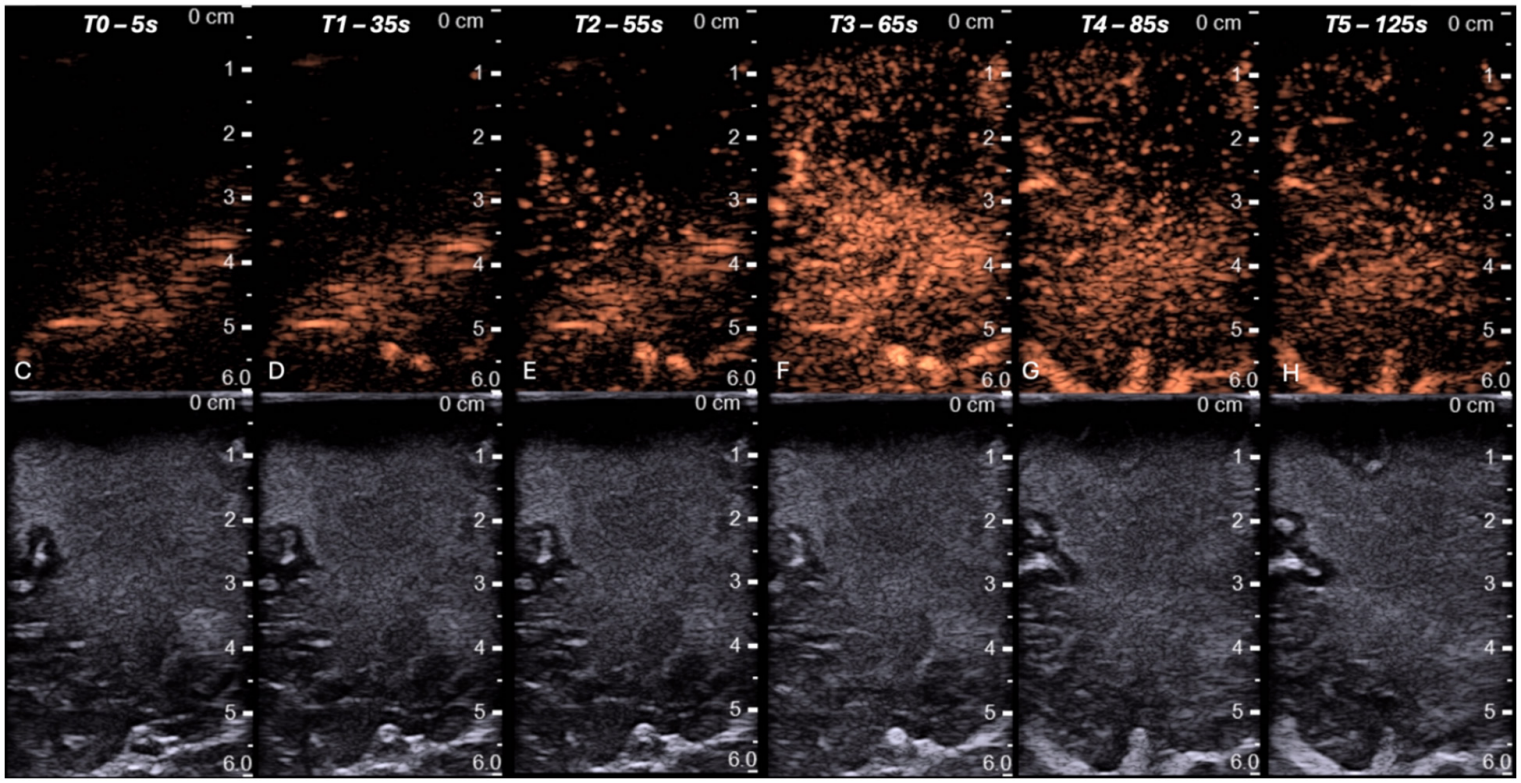
Intraoperative CEUS images acquisition at predefinited timepoints. We note the slow uptake phase beginning after 35 s and peaking after 1 min from microbubbles injection. The enhancement appears vivid and long-lasting, with the washout phase after roughly 2 min.

Based on the patient's medical history, the primary diagnostic suspicion was cerebral relapse of the lymphoma; however, differential diagnosis included diffuse glioma, primary central nervous system (CNS) lymphoma, and lastly inflammatory or demyelinating white matter disease. Routine blood tests were unremarkable except for neutrophilia and mild lymphocytopenia, likely related to ongoing high-dose corticosteroid therapy (dexamethasone 8 mg i.v three times daily) administered in the preceding days. Given the need for adequate tissue sampling for histopathological, microbiological, and molecular analyses, an open surgical biopsy was preferred over stereotactic needle biopsy. High-dose corticosteroid exposure was also considered a potential cause of false-negative results in the case of lymphoma, although the data on this matter are contradictory (Ghoche et al., 2025; Tosefsky et al., 2024). A preoperative lumbar puncture was performed for cerebrospinal fluid (CSF) analysis. Intraoperatively, ultrasound (IOUS) was performed using a high-frequency (7–12 MHz) linear probe with sterile coupling gel. Ultrasound imaging was obtained before and after dural opening in two orthogonal planes. B-mode settings were optimized intraoperatively, with adjustment of gain, depth, and focal zone to enhance lesion-to-parenchyma contrast. The focal zone was positioned at the level of the lesion. Baseline B-mode imaging demonstrated a homogeneous, hyperechoic, poorly marginated area within the right frontal white matter, with preservation of cortical architecture (Figure 2). Subsequently, 5 mL of sulfur hexafluoride microbubbles (SonoVue^®^, Bracco Imaging, Italy) were administered intravenously, followed by a 5 mL saline flush. Contrast-specific imaging software was used to record enhancement dynamics for 130 s, and all images were digitally archived. Contrast-enhanced ultrasound (CEUS) was performed using a low mechanical index technique (MI < 0.1) with contrast-specific imaging software in real-time dual mode. It revealed a distinctive enhancement pattern characterized by a delayed uptake phase beginning approximately 35 s after injection and peaking at around 55–60 s. The enhancement appeared intense, vivid, and relatively long-lasting, with washout occurring after 2 min. Notably, the deeper portions of the lesion exhibited earlier and more pronounced contrast uptake, suggesting a gradient in perfusion within the affected white matter (Figure 3). Intraoperative flow cytometric analysis of fresh tissue samples did not demonstrate any abnormal immunophenotype consistent with lymphoma recurrence. CSF analysis showed a non-specific increase in white blood cells, while real-time polymerase chain reaction confirmed the presence of JCV DNA (8,616.6 IU/mL). Tissue sample was finally processed with hematoxylin–eosin staining and immunohistochemistry tested for several antigens, such as CD20, CD3, CD5, Bcl2, Bcl6, CD138, IDHR132H, GFAP, Olig2, NFP, Cromogranin, and Ki67. Histopathological examination demonstrated extensive areas of demyelination involving the white matter, characterized by loss of myelin with relative preservation of axons. Numerous foamy macrophages containing myelin degradation products were observed, consistent with active demyelination. The astrocytic component showed marked reactive changes, including enlarged, atypical astrocytes with hyperchromatic and pleomorphic nuclei. In addition, oligodendrocytes exhibited nuclear enlargement with a ground-glass appearance, suggestive of viral cytopathic effect. These findings, in conjunction with the detection of JCV DNA in the CSF, were consistent with a diagnosis of PML.

The patient was discharged on the fourth postoperative day, in good clinical and neurological condition, and able to walk independently. He therefore began treatment with pembrolizumab and neuroradiological follow-up with contrast-enhanced MRI every 2–3 months. Even if PML prognosis is dismal, at the most recent follow-up, approximately 8 months after the diagnosis of PML, the patient is still alive and in good condition, with a Karnofsky performance status of approximately 90.

## Discussion

PML remains a significant diagnostic challenge. Although MRI is essential for early detection, its findings are not pathognomonic and may overlap with diffuse gliomas, primary CNS lymphoma, or treatment-related leukoencephalopathy. Specifically, when compared with other entities in the differential diagnosis, the ultrasound findings observed in this case show partial overlap but also notable differences. Diffuse gliomas, especially high-grade gliomas, typically demonstrate heterogeneous echogenicity and variable contrast enhancement, often with earlier uptake reflecting neovascularization. In contrast, treatment-related changes such as radiation necrosis may present with heterogeneous or peripheral enhancement patterns (Dixon et al., 2022; Yeole et al., 2020; Del Bene et al., 2018; Prada et al., 2014). Data on CEUS characteristics of demyelinating diseases remain limited; however, they are generally not associated with the rapid, intense enhancement typical of highly vascularized neoplasms. In our case, the delayed and relatively persistent enhancement pattern may suggest altered perfusion dynamics rather than neoplastic angiogenesis, although this interpretation remains speculative and requires further validation. In this context, establishing a definitive diagnosis often requires integration of clinical, radiological, virological, and histopathological data (Wu et al., 2011). To the best of out knowledge, the ultrasonographic characteristics of PML have never been described to date. In our case, IOUS demonstrated an homogeneous, hyperechoic, poorly marginated, a pattern that may resemble diffuse glioma, while CEUS revealed a delayed and relatively persistent contrast enhancement pattern, with a center-to-periphery gradient of contrast uptake. These findings should be interpreted with caution. The observed enhancement pattern may reflect altered microvascular perfusion within demyelinated white matter; however, this interpretation remains purely speculative. Given the single-case nature of this report, no definitive conclusions can be drawn regarding the underlying mechanisms or the reproducibility of these imaging characteristics. From a practical perspective, IOUS and CEUS may provide adjunctive intraoperative information in selected cases, particularly when lesion characterization is uncertain and tissue sampling is required. Nowadays, IOUS is widely used in brain tumor surgery (Prada et al., 2022; Padayachy and Prada, 2024), but its application in multifocal or demyelinating disease is limited by the fact that these conditions are often diagnosed using stereotactic needle biopsy. In our case, IOUS and CEUS imaging contributed to targeting areas for biopsy, although it did not allow reliable differentiation between demyelinating and neoplastic pathology. Therefore, these techniques should be considered supportive rather than diagnostic. The diagnostic complexity of neurological presentations in oncologic patients has been increasingly recognized, as highlighted in recent literature (Martinelli et al., 2024), which emphasizes the broad differential diagnosis and the potential for misinterpretation of imaging findings in this population. Our case further illustrates the importance of maintaining a high index of suspicion for non-neoplastic etiologies, even in patients with a strong oncologic history. We are aware that IOUS role will remain limited in routine surgical practice by the preferred use of stereotactic biopsy in diffuse lesions. Nevertheless, where open biopsy is indicated, IOUS and CEUS may offer real-time guidance and potentially improve sampling accuracy. Overall, the imaging features described in this report should be considered hypothesis-generating. Further studies, ideally involving larger patient cohorts, are needed to determine whether specific ultrasound patterns can be consistently identified in PML and whether they may really contribute to improved intraoperative decision-making.

This report provides among the first description of IOUS and CEUS features in PML. The combination of homogeneous hyperechogenicity and delayed, persistent contrast enhancement may help distinguish PML from neoplastic lesions, guide biopsy, and improve intraoperative diagnostic confidence.

## Data Availability

The datasets presented in this article are not readily available because of ethical and privacy restrictions. Requests to access the datasets should be directed to the corresponding author.
